# Patient safety risk associated with synchronous telehealth: A scoping review

**DOI:** 10.1371/journal.pone.0336992

**Published:** 2025-12-16

**Authors:** Juliana Salomão Rocha de Oliveira, Rafael Saad Fernandez, Mônica Rossatti Molina, Ana Carolina P.N. Pinto, César Ramos Rocha-Filho, Tiago M. Ferreira, Stela Verzinhasse Peres

**Affiliations:** 1 Departament of Philanthropy, Hospital BP - A Beneficência Portuguesa de São Paulo, São Paulo, Brazil; 2 Department of Research and Education, Hospital BP - A Beneficência Portuguesa de São Paulo, São Paulo, Brazil; 3 Iberoamerican Cochrane Centre, Barcelona, Spain; 4 Primary care physician. Sistema Único de Saúde (SUS), São Paulo, Brazil; Shiraz University of Medical Sciences, IRAN, ISLAMIC REPUBLIC OF

## Abstract

**Objective:**

We aimed to analyze the risks associated with patient safety in synchronous telehealth.

**Methods:**

Scoping review with search in 3 databases, Medical Literature Analysis and Retrieval System Online (Medline), via PubMed®, Embase® via Elsevier®, and the Cochrane Database of Systematic Reviews, recovering evidence from inception until September 4^th^, 2024. Eligible reviews investigated patient safety concerns arising from real-time interactions between healthcare professionals and patients through information and communication technologies (ICT), including telephones and videoconferencing tools. We included systematic reviews examining real-time telehealth interactions between healthcare professionals and patients, addressing safety concerns. We followed standard Joanna Briggs Institute methods for conducting the scoping review.

**Results:**

A total of 3,641 titles and abstracts were retrieved, and after screening, 15 systematic reviews were included, encompassing 315 studies. These reviews addressed various patient populations, healthcare settings, and telehealth interventions, including virtual consultations, telepharmacy, and telerehabilitation. All 15 reviews reported patient safety risks associated with telehealth, the most frequently reported concern was the patient’s experience, highlighted in 53.3% (n = 8) of the included studies. Additional concerns involved user knowledge gaps and the lack of safety criteria in evaluation protocols. These risks were categorized into five domains: patient experience, safety in prescribing medication, effective communication, training and education, and patient identification.

**Conclusion:**

This scoping review provides evidence that, although telehealth offers valuable alternatives for healthcare delivery, all evidence highlights specific patient safety risks that require attention. Further research is essential to better understand and mitigate these risks. Strategic investments in education, training, and structured implementation are critical to minimizing adverse events and enhancing patient safety in synchronous telehealth.

## Introduction

Telehealth, as defined by the World Health Organization (WHO), encompasses activities, systems, and health services conducted remotely by professionals using Information and Communication Technologies (ICT). Its objectives include providing diagnosis, promoting global health, facilitating the treatment and prevention of diseases and injuries, and advancing health research and education [1].

In 2016, the WHO highlighted the risk of new errors arising from the adoption of telehealth practices, emphasizing the need for telehealth projects to incorporate robust forecasting and planning strategies to effectively address potential harms issues. Addressing patient safety risks in telehealth involves navigating complex and dynamic processes, as these risks are systemic, affecting not only healthcare professionals but also the environment and organizational context [2].

Understanding how errors propagate through the care chain is essential for addressing the specific risks posed by information and communication technologies (ICT) in healthcare. As telehealth continues to expand rapidly, ensuring patient safety becomes increasingly critical. However, despite its central relevance, patient safety remains an underexplored aspect in telehealth, with notable gaps in regulatory frameworks and a lack of comprehensive guidelines in the literature regarding its implementation and monitoring [3]. A systematic review published in 2014 identified the risks associated with patient safety in telehealth within a home care context. However, the present review differs by investigating the risks related to patient safety in telehealth delivered through synchronous care, regardless of the setting [4].

Thus, this research aims to analyze the risks associated with patient safety in real-time interaction between healthcare professionals and patients, known as synchronous telehealth. The following review question guided our study: What are the patient safety risks associated with synchronous telehealth?

## Methods

### Study design

We conducted a scoping review of systematic reviews, following the guidelines of the Joanna Briggs Institute (JBI) [5]. This approach enabled a rapid comprehensive exploration of the existing evidence, with a narrative synthesis aimed at mapping key concepts and identifying gaps in literature. The review question and eligibility criteria were established in collaboration with method specialists and decision-makers. Reporting was guided by the Preferred Reporting Items for Systematic Reviews and Meta-Analyses Extension for Scoping Reviews (PRISMA-ScR) (S1 File) [6].

### Review question

The following review question guided our study: What are the patient safety risks associated with synchronous telehealth?

### Search strategy

An experienced review specialist (ACPNP and CRF) designed and conducted systematic searches of the literature in three major databases: Medical Literature Analysis and Retrieval System Online (Medline), via PubMed®, Embase® via Elsevier®, and the Cochrane Database of Systematic Reviews. These databases were selected for their comprehensive coverage of clinical and systematic review literature.

The search strategy was based on a combination of health descriptors, entry terms, and free-text vocabulary related to patient safety, telehealth, and systematic reviews. We combined terms for each concept using the Boolean operator “OR” and linked different concepts with “AND”. A list of keywords and synonymous terms used to capture potential studies across the resources is summarized in Chart 1. No restrictions were applied on publication dates, covering all evidence published from inception to September 4^th^, 2024. The full search strategy template for each database is provided in S1 Table.

**Chart 1:** List of keywords and synonyms generated as search terms.

Patient Safety		Telehealth	
Patient Safety	Never Event	Telemedicine	Teleconsultation
Safety Management	Near Miss	Telehealth	Telecommunication
Medical Error	Patient Harm	Tele-referral	Video Consultation
Medical Mistake	Misdiagnosis	Telemanagement	Mhealth
Medical Incident	Medication Error	Telecare	Mobile Health
Incident	Therapeutic Error	Telepharmacy	Ehealth
Medical Event	Inappropriate Prescribing	Telenursing	Remote Consultation
Wrong Procedure	Inappropriate Prescription	Telehomecare	Remote Care
		Tele Home Care	Remote Diagnosis
		Telehealthcare	

In addition to electronic searches, we (ACPNP and CRF) screened the references of eligible studies and searched for final publications of reports initially found as protocols or conference abstracts to capture additional studies that may have been missed in the original search.

### Eligibility criteria

The eligibility criteria for this scoping review are summarized in **Table 1**. Thus, we included studies that investigated the types of safety concerns in the real-time interaction process between healthcare providers and patients through any ICT. For the present study, ICT was broadly defined as a set of technological resources, such as telephones and videoconferencing tools, used by health professionals to support patient care.

**Table 1:** Eligibility criteria for study inclusion and exclusion.

Criteria	Inclusion Criteria	Exclusion Criteria
Type of study	Systematic reviews with clearly defined research questions, explicit eligibility criteria, and reproducible methodology for searching, selecting, appraising, and synthesizing evidence.	Studies without critical appraisal of included studies.
Technology use	Use of ICT (Information and Communication Technology) to support patient care, including telephones and videoconferencing tools.	Studies involving mixed samples (synchronous + asynchronous) without reporting separate results for synchronous care.
Interaction type	Focus on real-time (synchronous) interaction between healthcare professionals and patients.	Use of asynchronous technologies.
Population	Individuals requiring clinical medical care, regardless of age.	Technology used only for screening or triage.
Concept	Patient safety risk.	–

Systematic reviews were exclusively included in this scoping review with the aim of mapping consolidated knowledge within the secondary literature, thereby facilitating the identification of well-established areas and persistent gaps. This approach also aimed to ensure a higher methodological quality of the included studies while avoiding the duplication of data already synthesized in primary research. We did not limit the review to specific outcomes, allowing the inclusion of studies that assessed various aspects of patient safety, such as adverse events, medical errors, breaches in privacy, technology failures, or communication breakdowns. Studies that used technology solely as a screening tool or included mixed populations (e.g., synchronous, and asynchronous) without reporting separate results for synchronous participants were excluded. There were no restrictions on the design of primary studies included in the reviews or year of publication.

We restricted our review to full-text articles published in English, Spanish, or Portuguese, as these were the languages of expertise for the authors.

### Selection process

The articles recovered through the search strategy were imported into the web-based bibliographic manager Rayyan QCRI [7]. After duplicates were removed, two review authors (ACPNP and CRF) with expertise in systematic reviews independently screened all titles and abstracts, followed by a full-text review. Throughout the entire process, a third reviewer (TGM) resolved any discrepancies.

### Data extraction

One review author (ACPNP) extracted data from the included studies into pre-defined standardized forms using Microsoft Excel 365® (Microsoft Corporation, Redmond, WA, USA). A second review author (CRF) cross-checked the data extraction for completeness and accuracy. Disagreements were resolved through discussion and consensus or with the help of a third reviewer (TGM). The overlap of primary studies in the included systematic reviews was considered narratively, as further statistical analysis was not within the scope of this review.

### Synthesis of the results

The results were summarized narratively (ACPNP, CRF and JSRO) without exploring possible causes of heterogeneity. These summaries were consulted to identify the most appropriate way of presenting the results and then were sent to the team for review and discussion (ACPNP, CRF, JSRO, SVP, MRM, RSF). This approach was chosen due to the variability in study designs and outcomes across the included reviews. We applied a ‘descriptive‐analytical’ method using a common analytical framework, as outlined by Arksey and O’Malley (2005) [8] to categorize and interpret the findings.

## Results

### Results of the search

**Fig 1:**
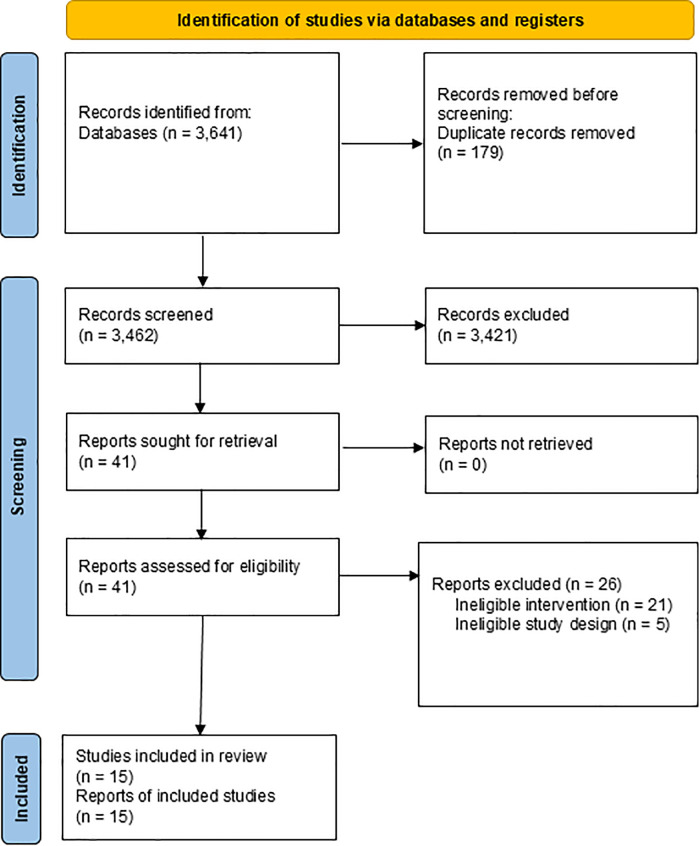
Identification of studies via databases.

Our search initially retrieved a total of 3,641 titles and abstracts. After removing duplicates, 3,462 abstracts were screened. Of these, 3,421 studies were excluded, justification can be found in S2 Table, leaving 41 studies for further scrutiny. In the end, we included 15 systematic reviews (**Fig 1**).

### Characteristics of included systematic reviews

We included 15 systematic reviews published between 2013 and 2024, covering a total of 315 studies. The number of studies in these reviews ranged from 4 to 80, with most reviews including between 10 and 30 studies. All were traditional systematic reviews of primary studies, except for three: a) one included both primary studies and reviews [4]; b) others included systematic overview of reviews [9,10]. Most reviews focused on randomized controlled trials (RCTs), supplemented by cohort and observational studies. A few also incorporated qualitative or mixed-methods studies **Table 2**. These reviews covered research conducted in a range of countries, reflecting the global scope of synchronous telehealth.

**Table 2:** Characteristics, methodological appraisal, and safety results in telehealth systematic reviews.

Study	Search details	N of included studies	Population/Setting	Interventions	Methodological quality/Risk of bias in included studies (instrument)	Safety results	Certainty of Evidence (instrument)	Effectiveness of Intervention
Grygorian et al. (2024)	February 2023, PubMed, Cochrane Library, Web of Science	19 studies (11 RCTs, 8 cohort studies)	Patients undergoing abdominal surgery	Telemedicine interventions (e.g., video calls, mobile apps, remote monitoring)	Limited due to lack of blinding and selection of participants; RCTs: PEDro Scale; Non-RCTs: ROBINS-I Tool	Concerns related to patient experience	Low certainty due to heterogeneity and limited data in studies (no instrument used)	Telemedicine reduced hospital readmissions and emergency visits, but no significant increase in complications
Campbell et al. (2023)	June 2022, MEDLINE, Embase, HMIC, PsycINFO, CINAHL, Cochrane	30 studies: 14 retrospective cohorts, 6 cross-sectional, 4 quasi-experimental, 3 RCTs, 2 cohort studies, 1 cluster RCT	Primary care settings, various conditions, global	Virtual consultations (telephone, video)	Moderate to high risk of bias (Mixed Methods Appraisal Tool)	Issues in effective communication	Low to very low certainty due to variability across studies and few studies (no instrument used).	Virtual consultations conducted via videoconference may have similar diagnostic accuracy to face-to-face for most conditions (no important risk of misdiagnosis)
Darley et al. (2022)	January 2010 to February 2022, Ovid MEDLINE, EMBASE, Web of Science, Scopus	63 studies (33 quantitative, 12 qualitative, 18 mixed methods)	Primary care settings in 9 high-income countries	Online consultations (OCs) in primary care	67% studies rated “good” (Mixed Methods Appraisal Tool (MMAT))	Combined issues in communication and patient experience	From high to low depending on the findings (GRADE CerQual approach)	No quantitative evidence of negative impacts on patient safety, qualitative perceptions varied regarding patient safety
Gleeson et al. (2022)	June 2021, Embase, Medline via PubMed, English language articles from 2000 onward	15 studies (11 cross-sectional, 2 retrospective, 1 case series, 1 controlled before-and-after study)	Primary care, mostly in the U.S., with some studies from the U.K., Sweden, and Finland	Electronic prescribing and telemedicine in primary care	Acceptable quality, assessed with CASP Checklist	Concerns regarding medication safety and patient experience	Limited by heterogeneity (no instrument used)	Common incidents include ‘wrong label/instruction’ and ‘wrong dose/strength/frequency’;
Vieira et al. (2022)	March 2022, MEDLINE, Cochrane, EMBASE, LILACS	6 RCTs	Patients with COVID-19 and post-COVID conditions	Telerehabilitation (breathing exercises, aerobic and strength exercises)	Some concerns to high risk of bias (Cochrane Risk of Bias 2.0)	Gaps in training and professional education	Low to moderate certainty of evidence, mainly due to risk of bias and imprecision in estimates (GRADE approach)	Low incidence of adverse events associated with telerehabilitation
Corso et al. (2021)	January 2021, MEDLINE, CINAHL, Embase, Cochrane Central	9 RCTs	Adults with musculoskeletal conditions, primary and secondary care settings	Nonpharmacologic interventions (e.g., exercise, education) delivered via telehealth (8 telephone, 1 videoconference)	Low risk to some concerns (Cochrane Risk of Bias 2)	Patient experience-related concerns;	Low to moderate due to lack of blinding and imprecision in estimates (GRADE approach)	Telehealth interventions were as effective as in-person care
Haveland and Islam (2021)	2015-2020, Medline, CINAHL, Google Scholar, UNE Library	21 (48% quantitative, 33% qualitative, 19% mixed methods)	Telehealth care models in rural and regional settings	Telehealth consultations, remote care services	Generally limited, Generic critical appraisal tool by Woolliams et al., CASP checklists	Risks related to training, patient experience, and medication safety; diagnostic errors reported	Limited by variability in studies (no instrument used)	Comparable outcomes to in-person care
Kia et al. (2021)	September 2020, PubMed, Scopus, Web of Science, Cochrane Library, Google Scholar	18 studies (12 with high risk of bias, 9 with real-time video conferencing, 8 on cost reduction)	Telemedicine in emergency department settings	Various telemedicine technologies: real-time video conferencing, telemonitoring, store-and-forward	High risk of bias (ROBIS tool used)	Communication issues; technical failures, maintenance challenges, and privacy concerns	Limited by high bias and heterogeneity (no instrument used)	Cost reduction. Improvements in resource utilization and operational efficiency.
Pathak et al. (2021)	August 2020, PubMed, Embase, Scopus	6 cross-sectional studies	Community pharmacies, rural and underserved areas	Telepharmacy services in community settings	High risk of bias (RoBANS tool used)	Medication safety risks; high error rates in telepharmacies and conflicting patient experience	Not definitive due to high risk of bias; stronger study designs needed for better evidence (no instrument used)	Medication adherence similar to conventional care
Svendsen et al. (2021)	2015 to March 2021, PubMed, Embase, Web of Science, Cochrane Library	4 (3 RCTs and 1 cohort study)	Various eHealth settings (Denmark)	eHealth as a communication tool between patients and healthcare providers	Medium to low risk of bias (SIGN methodology checklist)	Risks in communication, training, patient experience and patient identification	Limited due to small number of studies and variability in designs (no instrument used)	Improvements in management of hypertension and weight loss
Han et al. (2020)	February 2020, MEDLINE, Embase, HMIC, PsycINFO, and CINAHL	12 (1 RCT and 11 cohort studies)	Primary health care settings, global	Remote consultations (telephone, text-based, video)	Fair to moderate quality, assessed with National Heart, Lung, and Blood Institute tool (for observational studies) and Cochrane RoB tool (for RCTs)	Risks associated with medication safety	Inconsistency across studies limits conclusions (no instrument used)	Mixed results in antibiotic prescribing rates
Rush et al. (2018)	April 25, 2018, Medline, CINAHL, Embase	8 (6 RCTs, 1 Retrospective chart reviews and 1 Quantitative Repeated Measures study)	Various healthcare settings (e.g., stroke, psychiatric disorders, smoking cessation)	Videoconference vs Telephone consultations	Mixed quality, McGill Mixed Methods Appraisal Tool (MMAT)	Risks associated with medication safety	Limited by small sample sizes, heterogeneity and short follow-up periods (no instrument used)	Videoconference resulted in fewer medication errors, higher diagnostic accuracy, and improved decision-making accuracy compared to telephone. Comparable patient outcomes, but some differences in provider outcomes
Mackintosh et al. (2016)	December 2015, MEDLINE, EMBASE, CINAHL, Cochrane, HTA, Web of Science	2 controlled before-after studies	Critical care settings (ICU)	Telemedicine with 24/7 clinical decision support in ICUs	High risk of bias (EPOC guidelines)	Issues related to patient experience	Limited by poor methodological quality and few data (no instrument used)	Reduced ICU and hospital mortality; better adherence to best practices
Guise et al. (2014)	November and December 2013. Databases: Medline, CINAHL, ISI Web of Knowledge, Academic Search Premier (ASP), Scopus, Science Direct. English language	22 (11 qualitative studies, 3 case studies, 3 mixed-method studies, 3 quantitative studies, 2 systematic reviews)	Homecare services using telecare, with adults aged 18+ in home settings	Telecare systems used in homecare, including monitoring devices, video consultations, etc.	Acceptable quality (CASP tools for qualitative, review, and quantitative studies).	Communication risks: 11 safety incidents related to workflow, technology, and knowledge gaps	Limited by study design and heterogeneity (no instrument used)	Not reported
McLean et al. (2013)	January 1997 – November 2011, Cochrane Library, MEDLINE, EMBASE, LILACS, IndMed, PakMed	80 systematic reviews	Various telehealthcare models (synchronous and asynchronous)	Telehealthcare including monitoring and patient feedback	Generally moderate (CASP tool for systematic reviews)	Deficiencies in training and patient experience; patient safety rarely addressed in telehealth evaluations	Limited by heterogeneity and short follow-up periods (no instrument used)	Reduction in hospital and ICU mortality; improvements in best practice adherence, but limited by high bias

The study by Mackintosh et al. (2016) [11] reported data exclusively from the United States. The study by Han et al. (2020) [12] included reviews from Norway, Scotland, the United States, and Denmark. One review included studies from nine countries: the United States, Australia, Kenya, Canada, the United Kingdom, Japan, Singapore, New Zealand, and Sweden [13]. Another review also covered multiple countries, such as the United Kingdom, Sweden, New Zealand, Spain, Canada, Norway, and Denmark [14]. Rush et al. (2018) [15] included Germany, the United States, Northern Ireland, and China. Gleeson et al. (2022) [16] examined research from Sweden, the United States, England, and Finland. In another study, the included countries were Germany, China, Colombia, Malaysia, and the United States [17]. Pathak (2021) [18] focused on studies conducted in the United States. Haveland and Islam (2022) [19] brought together studies from a broad range of countries, including the United Kingdom, Australia, the United States, Norway, Denmark, Spain, and Canada. Finally, Vieira et al. (2022) [20] reported data from Spain, China, Turkey, and Brazil.

The United States was cited in 9 (60%) reviews, followed by 6 Norway, 5 the United Kingdom and Canada each, and 4 Sweden. Other countries represented included Germany, China, Spain, Denmark, Finland, Colombia, Malaysia, Northern Ireland, Brazil, Turkey, Kenya, Japan, Singapore, and New Zealand.

### Populations and settings studied

Regarding the populations analyzed, three reviews focused on specific clinical groups: Vieira et al. (2022) [20] included patients with COVID-19 and post-COVID conditions, Grygorian et al. (2024) [21] focused on patients undergoing abdominal surgery, and Corso et al. (2022) [22] examined adults with musculoskeletal disorders. The age distribution within studies was not consistently reported, limiting further stratification by age group.

In terms of the care settings, primary care was the most frequently reported environment, appearing in 5 reviews (33%) [9,11–14]. Other settings included homecare services in 2 reviews [4,9], emergency departments in 1 [10], and community pharmacies in 1 [18]. The use of telehealth in rural or underserved regions was examined in 3 reviews (20%) [4,17,19].

### Telehealth interventions

Among the 15 studies included in this review, the most frequently analyzed intervention was synchronous videoconferencing, appearing in 12 reviews (80%) [4,9,10,12–16,19–22]. In comparison, remote patient monitoring systems were discussed in 5 reviews (33%) [4,9–11,21]. In addition, telephone-based care was reported in 4 reviews (27%) [12,13,15,22], and 4 reviews [10,13,18,22] described text-based interventions, such as asynchronous messaging or mobile applications. Furthermore, telepharmacy services, including remote prescription verification and counseling, were described in 1 review [19], and telerehabilitation programs for musculoskeletal and post-operative care were addressed in 1 review [21].

### Safety results and concerns

Safety-related outcomes exhibited notable variation across the reviews, as described below. Identified risks were categorized into five areas: patient experience, medication safety, communication, training, and patient identification.

Concerns related to patient experience were cited in 60% (n = 9) of the reviews [9,11,14,16–19,21,22]. Following this, issues involving medication safety were reported in 6 reviews (40%) [12,15–19]. Communication-related risks were examined in 5 reviews (33%) [4,10,13,14,17] and the same number of reviews addressed concerns related to training and education [4,9,17,19,20]. In contrast, only 1 review (7%) focused on patient identification risk.

The most commonly reported incidents were wrong labels, instructions, or medication doses [16]. Adverse events associated with tele-rehabilitation programs have also been found [20]. The studies included in this scoping review reported results comparing telehealth and face-to-face care in different scenarios. No significant differences were found [13,18,19,22], although additional benefits such as cost reduction, improved resource utilization, and operational efficiency were noted in some reviews [10].

When comparing modalities, videoconferencing was associated with fewer medication errors, greater diagnostic accuracy, and more consistent clinical decision-making than telephone consultations. However, no substantial differences were observed in patient outcomes [16]. Detailed results are provided in **Table 2**.

## Discussion

This scoping review identified patient safety risks associated with telehealth services. The most frequently reported concern was the patient’s experience, highlighted in 60% (n = 9) of the included studies [9,11,14,16–19,21,22]. The second most cited risk was medication safety, appearing in 40% (n = 6) of the articles [13,16–20]. Other relevant risks included communication failures, patient identification, and gaps in training related to telehealth practices. These findings underscore the complexity of ensuring safe, high-quality care in virtual environments and point to priority areas for system-level improvements.

Given these challenges, it is essential to situate discussion within the broader global movement for patient safety. In 2004, the WHO created the Global Alliance for Patient Safety, which drew up the “Patient Safety Goals” (PSG) to guide global efforts to reduce patient harm [23].

The growing use of telehealth expands access to health services, as it reduces costs and travel time [24]. However, the use of ICT can also introduce new risks to the safety of care [4]. Problems with health IT have been widely documented, such as system errors, poor user interfaces, and lack of proper training, can disrupt the care process and lead to delays in decision-making or errors in clinical tasks, ultimately increasing the likelihood of patient harm [25].

The relevance of the patient’s experience is increasing, requiring physical and digital services to be concerned with users’ overall perception of the care offered. This includes understanding patients’ needs and expectations. Health services must find ways to meet these expectations by offering digital means for health promotion, prevention, rehabilitation, or treatment [26].

For safe and reliable assistance, care results must be aligned with better outcomes, cost reduction, promotion of favorable experience and good patient acceptance. In some cases, the convenience of digital care is linked to commuting, making telehealth a preferred option [14,19,21].

In this context, McLean et al. (2013) [9] found that rural populations tend to be more satisfied with remote care, preferring it to the physical model, and Pathak et al. (2021) [18] observed greater satisfaction in telepharmacies when compared to traditional pharmacies.

Despite the risks, such as limitations in digital diagnosis and treatment without physical attendance, Svendsen et al. (2021) [17] reported that patients and doctors positively evaluated the safety and effectiveness of teleconsultation. In contrast, other studies did not find conclusive evidence. Corso et al. (2022) [22] found no difference in satisfaction between virtual and face-to-face care, while Mackintosh et al. (2016) [11] highlighted the need for more research into patient satisfaction.

The literature highlights, as a safety strategy, the importance of selecting an appropriate group of patients for telehealth. This process of carefully selecting patients for whom telemedicine is most appropriate is fundamental to guaranteeing patient safety and the provision of high-quality care. Providers must identify patients individually, with the active participation of each one, and make additional resources available if Internet access limitations represent barriers to care [27]. As noted in one of the studies included in our review, this strategy is particularly relevant in the context of chronic disease management, where telehealth has shown satisfactory outcomes [9].

In addition, the positive outlook of healthcare professionals appears to influence patient engagement positively [11,19]. Therefore, these findings suggest that the patient’s experience using telehealth tends to be positive. Providers should adopt appropriate patient selection criteria and maintain a patient-centered service that accounts for the limitations of virtual care.

In addition to patient experience, another central concern in this review relates to the safety of medication prescription in telehealth, reported in 40% (n = 6) of the included studies [12,15–19]. In 2017, the World Health Organization launched the Global Patient Safety Challenge with the theme “Medication Without Harm,” reinforcing the international relevance of this topic [28].Rush et al. (2018) [15] observed that video calls showed a reduction in medication errors compared to telephone calls, suggesting that visual interaction may be a positive factor. This finding indicates that the absence of visual interaction makes clinical assessment difficult, increases the risk of prescribing errors, and may compromise safety.

The potential to improve safety in prescribing medications, especially in remote areas, was highlighted by Pathak et al. (2021) [18] who found that telepharmacy showed better performance compared to traditional pharmacies in the detection of medication errors and adherence to treatment. However, the authors highlighted the need for more robust research to minimize possible biases.

Similarly, this observation is consistent with findings by Omboni and Tenti (2019) [29], who emphasized the importance of investigating the impact of telepharmacy and its potential risks to patient safety.

Drug prescribing in remote and face-to-face environments has been investigated to identify possible differences and challenges associated with each modality. Han et al. (2020) [12], when comparing the dosage and choice of antibiotics, found no significant differences. However, the authors emphasized the importance of preventing antimicrobial resistance, a risk present in both models of care. Complementing this analysis, Svendsen et al. (2021) [17] warned of the dangers related to prescribing antibiotics, controlled drugs and drugs with potential side effects, highlighting the need for strict monitoring of prescriptions and possible drug interactions.

This is consistent with findings from Ray et al. [30] who found higher antibiotic prescribing in children seen via telemedicine than in person. Such evidence underscores the risks associated with antibiotic prescribing in remote care environments. These findings reveal that communication is not only a frequent source of risk but also a mediating factor in the safe use of medicines.

Furthermore, other studies have associated effective communication with the safe use of medicines. Two of the included reviews in this research [16,19] emphasized this relationship, especially in contexts where clear interaction between professional and patient is essential. Gleeson et al. (2022) [16], for example, identified language barriers as factors that can cause harm during the prescribing process, reinforcing the need for clarity and accuracy in communication.

While Haveland and Islam (2022) [19] highlighted the importance of effective communication in managing adverse drug reactions in cancer patients, further supporting its role as a critical component of patient safety. The aim of WHO was to strengthen systems to reduce medication errors, since unsafe practices related to medications are one of the main causes of preventable harm [31].

Beyond patient-centered strategies, another critical issue identified was effective communication, the third most cited concern. Our analysis revealed, 33% (n = 5) of the studies included addressed communication as a risk to patient safety [4,10,13,14,17]. The emphasis differed depending on the study, encompassing issues such as connectivity problems, unclear instructions, barriers to professional access, and concerns related to information management in virtual care [4,10].

In this scoping review, we identified the risk of information being lost during virtual consultations, compromising understanding of the clinical picture and potentially leading to incorrect diagnoses [14]. This finding aligns with existing literature, which highlights communication failure as one of the most frequent factors in the occurrence of incidents, a concern that has also been raised in external source. This makes effective communication a crucial goal in various healthcare scenarios [32,33].

Findings from the present review indicate that barriers to accessing or communicating with professionals, as well as risks to confidentiality in teleconsultations, such as leakage of sensitive information, unauthorized access and misuse of personal data, were frequently reported across the included studies [4,10]. These concerns are corroborated by external literature, which points out the fact that violations of privacy may affect patient trust in telehealth services [34]. Furthermore, such breaches may lead to ethical and legal impacts [35].

Previous studies have similarly emphasized that effective communication plays an important role in reducing the likelihood of adverse events, as it minimizes errors related to ambiguities or failures in the communication process [36].

Our results indicate the importance of selecting patients suitable for virtual interventions, such as those in need of chronic disease management or lifestyle modification [19]. Campbell et al. (2023) [13] showed that videoconference consultations can have similar diagnostic accuracy to face-to-face consultations for most conditions. Moreover, videoconferencing has been shown to outperform telephone consultations in terms of probability of errors, diagnostic accuracy, and decision-making, creating barriers to error prevention [15]. This is consistent with external findings showing that videoconferencing can be as effective as in-person visits in certain contexts [37].

Although communication brings benefits, it can also harm patient safety [17]. These results align with prior research, wich indicates that risks can be mitigated when they are considered during the implementation of the service [1,16]. Therefore, safety measures should be included at all stages [16].

Within this framework, in-service training is a strategy adopted by health institutions that uses the work environment itself to enhance professional development and improve the quality of care, aligning practical knowledge with organizational culture [38].

In this context, the inclusion of the topic of “patient safety” in training and education programs within telehealth services was evaluated in 5 reviews (33%) [4,9,17,19,20]. In these reviews, it was recommended that professionals be formally prepared for remote care, ensuring that it is carried out without harm. The use of new technologies in healthcare brings risks to patient safety, requiring continuous educational updating [17].

Corroborating the findings of this scoping review, a recent study on telehealth training in allied health professions reinforces this need by showing that many programs lack pedagogical structure and consistent evaluation criteria, which compromises their effectiveness. It emphasizes that in-service training must be grounded in solid educational methodologies to ensure patient safety in remote care [39].

Additionally, the study by Garber and Gustin (2022) [40] reinforces that most healthcare providers do not receive formal telehealth training during their academic education and are expected to learn on the job. This educational gap highlights the strategic importance of in-service training as a compensatory mechanism for the safe and effective use of telehealth in clinical settings.

Moreover, both studies [39,40] indicate that structured educational approaches, such as online courses and simulation-based training, are significantly more effective than improvised or passive formats, like written instructions. These findings demonstrate that the type of training received influences not only provider competence but also adoption and satisfaction. Ultimately, it affects the quality of the patient’s experience.

Educational action should be broad, ranging from the use of technology and solving operational problems to interaction with patients and colleagues, increasing workers’ knowledge of telehealth [4,17,20].

Vieira et al. (2022) [20] evaluated patient safety and the occurrence of adverse events, classified as mild or moderate, highlighting the importance of trained professionals to adapt therapy with a view to patient safety.

The responsibilities inherent in telehealth work processes should be included in education actions [4,9]. Patients should also be trained to use telehealth safely and confidently [19].

In addition to training, accurate patient identification is a critical component of patient safety, recognized as the first goal proposed by the WHO [40].

This issue was explored in only one of the included reviews. Svendense et al. (2021) [17] highlighted potential risk to patient safety associated with identification failures during telehealth.

This finding is consistent with Riplinger et al. [41], who demonstrated that failure to perform it appropriately can compromise clinical decision-making, reduce treatment effectiveness, jeopardize patient outcomes and privacy.

Taken together, this evidence reinforces the concern that, in virtual settings, there is a risk of misidentification, which may lead to errors in orientation or diagnosis. Therefore, it is essential to implement verification processes to ensure that the patient engaged via videoconference or telephone is correctly identified. To support these safety measures, from accurate identification to effective communication, training emerges as a strategic tool within organizations.

This review has limitations related to the absence of standardized tools for assessing patient safety in telehealth. Different study populations were observed, including age group, diagnosis, setting, and country of origin, which may limit the comparability of findings and the depth of the analysis.

We can adapt good practice guidelines from the traditional care model to telecare. These practices may serve as a starting point for the digital environment, such as the creation of surveillance systems for reporting and monitoring adverse events.

Mapping the main adverse events is relevant not only for continuous process improvement, but also for the development of good practices in telehealth platforms and technologies [18]. Given the diversity of telehealth services and the different risk profiles, an approach based on PSG may constitute a possible approach. The adoption of surveillance actions would help to compose a profile of existing risks, allowing for the creation of more effective strategies to mitigate them.

There is also a lack of evidence on the intrinsic barriers to digital services that can lead to errors, such as connection difficulties, poor sound and image quality, and varying levels of digital literacy. To mitigate potential risks, it is necessary to map stages such as registration, information exchange, sending documents, transmitting test results, guidance and prescriptions [42].

In addition, it is necessary to engage and raise awareness among managers and healthcare professionals about patient safety in telehealth. These actors should develop training and continuing education actions for professionals and patients, reinforcing the importance of a transversal and interdisciplinary safety culture.

## Conclusion

This scoping review provides evidence that, while telehealth offers valuable alternatives for healthcare delivery, it also presents specific patient safety risks that warrant careful consideration. The most frequently reported risks were related to the patient’s experience and communication failures, followed by issues concerning medication safety, patient identification, and training gaps.

Corroborating findings from the included studies, this review reinforces the need to strengthen care processes to prevent safety incidents in virtual environments. Further research is essential to expand knowledge about these safety issues and to develop strategies to mitigate them. This discussion contributes to guiding future investigations, promoting structured production and dissemination of knowledge, and enhancing safety in telehealth services. To ensure a positive and secure impact of telehealth implementation, it is critical to invest in professional education, continuous training, and careful planning of care processes that reduce the likelihood of adverse events and promote reliable service delivery.

## Supporting information

S1 File S1 Checklist. (DOCX)

S1 Table Search Strategy. (PDF)

S2 Table Exclusion of studies. (PDF)

S1 Fig Identification of studies via databases. (TIFF)
