# Supplementary material for: Patient safety risk associated with synchronous telehealth: A scoping review
Source: PLoS One. 2025 Dec 16;20(12):e0336992. doi: 10.1371/journal.pone.0336992 (PMC12707622; doi:10.1371/journal.pone.0336992)
Supplement: S1 Table — (PDF) [file pone.0336992.s002.pdf]

S2 Supplementary Chart 2. Search Strategy. S2 Chart 2.

Study ID	Title	Reason for exclusion
Martiniuk et al. (2024)	A review of risks, adverse effects and mitigation strategies when delivering mental health services using telehealth	Ineligible study design - No critical appraisal assessment
Dat et al. (2023)	Telepharmacy: A Systematic Review of Field Application, Benefits, Limitations, and Applicability During the COVID-19 Pandemic	Ineligible study design - No critical appraisal assessment
Rosen et al. (2023)	Potential Harms Resulting From Patient-Clinician Real-Time Clinical Encounters Using Video-Based Telehealth: A Rapid Evidence Review: Rapid Review.	Ineligible study design - No critical appraisal assessment
Ewart et al. (2022)	Patient perspectives and experiences of remote consultations in people receiving kidney care: A scoping review	Ineligible study design - No critical appraisal assessment
Li et al. (2021)	Safety of video-based telemedicine compared to in-person triage in emergency ophthalmology during COVID-19.	Ineligible intervention – Triage
Chambers et al. (2019)	Digital and online symptom checkers and health assessment/triage services for urgent health problems: systematic review.	Ineligible intervention – Triage
Huang et al. (2019)	Efficacy and safety of telemedicine for blood glucose and pregnancy outcomes in gestational diabetes mellitus: A systematic review.	Ineligible intervention – not synchronous health care
Liddy et al (2019)	A Systematic Review of Asynchronous, Provider-to-Provider, Electronic Consultation Services to Improve Access to Specialty Care Available Worldwide.	Ineligible intervention – not synchronous health care
Rademacher et al. (2019)	Use of Telemedicine to Screen Patients in the Emergency Department: Matched Cohort Study Evaluating Efficiency and Patient Safety of Telemedicine.	Ineligible intervention – Triage

Borycki et al. (2018)	Methodologies for Improving the Quality and Safety of Telehealth Systems.	Ineligible intervention – not synchronous health care
Hassan (2018)	Tele-ICU and Patient Safety Considerations.	Ineligible intervention – not synchronous health care
Kaminsky et al. (2017)	Telephone nursing in Sweden: A narrative literature review	Ineligible intervention – Triage
Kim et al. (2017)	Problems with health information technology and their effects on care delivery and patient outcomes: a systematic review	Ineligible intervention – not synchronous health care
McConnochie et al. (2015)	Effectiveness and Safety of Acute Care Telemedicine for Children with Regular and Special Healthcare Needs.	Ineligible intervention – not synchronous health care
Salahuddin et al. (2015)	Classification of antecedents towards safety use of health information technology: A systematic review.	Ineligible intervention – not synchronous health care
Lyles et al. (2013)	Safety events during an automated telephone self-management support intervention.	Ineligible intervention – not synchronous health care
van-Velthoven et al. (2013)	Telephone delivered interventions for preventing HIV infection in HIV-negative persons	Ineligible intervention – not synchronous health care
Atherton et al. (2012)	Email for clinical communication between patients/caregivers and healthcare professionals	Ineligible intervention – not synchronous health care
Huibers et al. (2011)	Safety of telephone triage in out-of-hours care: a systematic review.	Ineligible intervention – Triage

Luxton et al. (2010)	Safety of telemental healthcare delivered to clinically unsupervised settings: a systematic review.	Ineligible study design - No critical appraisal assessment
St George et al. (2009)	How safe is telenursing from home?	Ineligible intervention – Triage
