# Supplementary figures and images for: Patient safety risk associated with synchronous telehealth: A scoping review
Source: PLoS One. 2025 Dec 16;20(12):e0336992. doi: 10.1371/journal.pone.0336992 (PMC12707622; doi:10.1371/journal.pone.0336992)

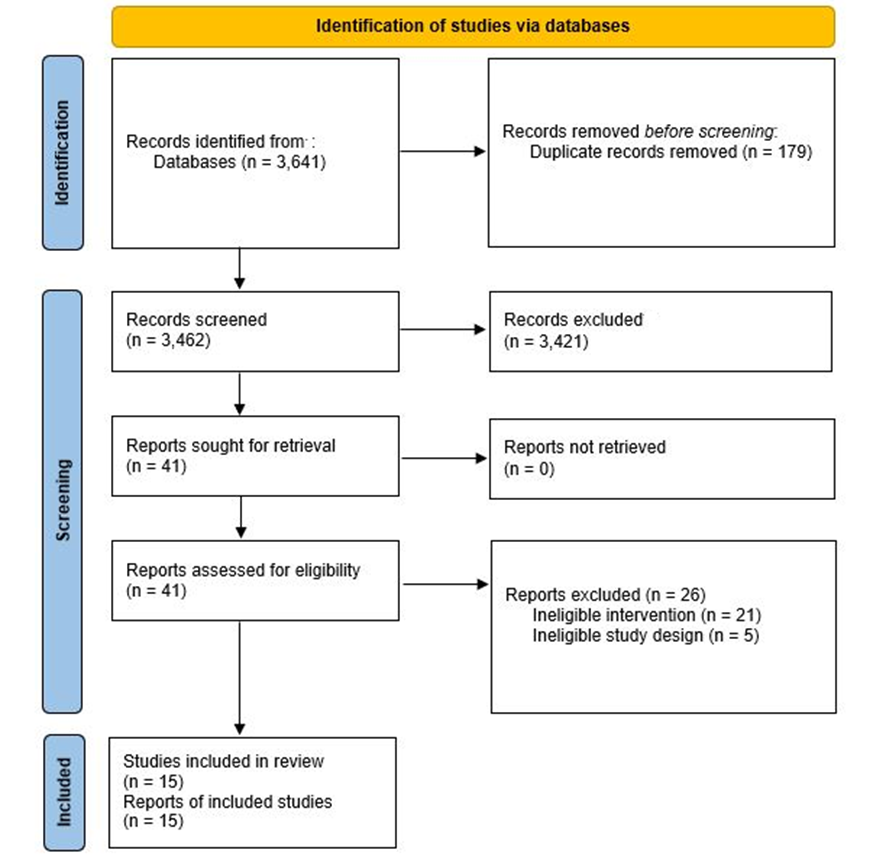

Supplement: S1 Fig — (TIFF) [file pone.0336992.s004.tiff]
